# Comparison of Fatty Acid Profiles in a Group of Female Patients with Chronic Kidney Diseases (CKD) and Metabolic Syndrome (MetS)–Similar Trends of Changes, Different Pathophysiology

**DOI:** 10.3390/ijms20071719

**Published:** 2019-04-06

**Authors:** Małgorzata Szczuko, Małgorzata Kaczkan, Arleta Drozd, Dominika Maciejewska, Joanna Palma, Anna Owczarzak, Natalia Marczuk, Przemysław Rutkowski, Sylwia Małgorzewicz

**Affiliations:** 1 Department of Biochemistry and Human Nutrition, Pomeranian Medical University in Szczecin, 71-460 Szczecin, Poland; arleta.drozd@gmail.com (A.D.); domi.maciejka@wp.pl (D.M.); palma.01.01@gmail.com (J.P.); 2 Department of Clinical Nutrition Medical University of Gdańsk, 80-210 Gdańsk, Poland; kaczkan@gumed.edu.pl (M.K.); anna.owczarzak@gumed.edu.pl (A.O.); sylwia.malgorzewicz@gumed.edu.pl (S.M.); 3 Department of Microbiology and Immunology, Pomeranian Medical University in Szczecin, 71-460 Szczecin, Poland; n.marczuk03@yahoo.pl; 4 General Nursery Department, Medical University of Gdańsk, Diaverum Hemodialysis Unit, 80-210 Gdańsk, Poland; przemyslaw.rutkowski@gumed.edu.pl

**Keywords:** fatty acids, chronic kidney disease, metabolic syndrome, nutrition

## Abstract

Fatty acid (FA) profiles in the plasma of patients with metabolic syndrome and chronic kidney disease (CKD) seem to be identical despite their different etiology (dietary mistakes vs. cachexia). The aim of this study was to compare both profiles and to highlight the differences that could influence the improvement of the treatment of patients in both groups. The study involved 73 women, including 24 patients with chronic kidney disease treated with haemodialysis, 19 patients with metabolic syndrome (MetS), and 30 healthy women in the control group. A total of 35 fatty acids and derivatives were identified and quantified by gas chromatography. Intensified elongation processes from acid C10:0 to C16:0 were noted in both groups (more intense in MetS), as well as an increased synthesis of arachidonic acid (C20:4n6), which was more intense in CKD. Significant correlations of oleic acid (C18:1n9), gamma linoleic acid (C18:3n6), and docosatetraenoate acid (C22:4n6) with parameters of CKD patients were observed. In the MetS group, auxiliary metabolic pathways of oleic acid were activated, which simultaneously inhibited the synthesis of eicosapentanoic acid (EPA) and docosahexaenoic acid (DHA) from alpha lipoic acid (ALA). On the other hand, in the group of female patients with CKD, the synthesis of EPA and DHA was intensified. Activation of the synthesis of oleic acid (C18: 1n9 ct) and trans-vaccinic acid (C18:1) is a protective mechanism in kidney diseases and especially in MetS due to the increased concentration of saturated fatty acid (SFA) in plasma. The cause of the increased amount of all FAs in plasma in the CKD group, especially in the case of palmitic (C16:0) and derivatives stearic (C18:0) acids, may be the decomposition of adipose tissue and the progressing devastation of the organism, whereas, in the MetS group, dietary intake seems to be the main reason for the increase in SFA. Moreover, in MetS, auxiliary metabolic pathways are activated for oleic acid, which cause the simultaneous inhibition of EPA and DHA synthesis from ALA, whereas, in the CKD group, we observe an increased synthesis of EPA and DHA. The higher increase of nervonic acid (C24:1) in CKD suggests a higher degree of demyelination and loss of axons.

## 1. Introduction

Fatty acids (FAs) in kidney diseases are sporadically described in the literature. It is known that the changes in the fatty acids of plasma contribute to lipotoxicity and the accumulation of excessive lipids in kidneys, which is a factor facilitating the development and progress of kidney disease, especially in the case of accompanying obesity and diabetic nephropathy (DN) [1]. This can also contribute to the development of chronic kidney disease (CKD), irrespective of pathology [2]. Lipotoxicity induced by saturated FAs (SFA), including palmitic (C16:0) and stearic (C18:0) acids, causes insulin resistance and cell death. Despite this, lipotoxicity seems to be an adaptive process that enables the podocytes, for a short time, to cope with increased levels of fatty acids (FA) [3]. However, when this buffering mechanism becomes overloaded, lipotoxicity together with insulin resistance and endoplasmic reticulum (ER) stress may lead to the death of a podocyte, which contributes to the progress of the disease [3,4]. ER stress was also noted in interstitium tubules and renal glomeruli in patients with DN [5]. Nonetheless, mono-unsaturated fatty acids (MUFA)—including oleic acid (C18:1 n-9), which is present in olive oil—prevent the death of podocytes, which, in turn, can prevent and/or delay the development of kidney disease [6]. Moreover, the data concerning patients with kidney disease show that an increasing amount of nervonic acid (C24:1) is a significant prognostic factor for death in the fifth stage of the disease [7]. According to Dołęgowska et al., treatment using haemodialysis results in an increased level of nervonic acid in patients [8]. The unbalanced metabolism of FA seems to be similar in patients with metabolic syndrome and diabetes [9,10]. Both diseases are associated with abnormal nutrition and lifestyle. In contrast, CKD is a debilitating disease that leads to malnutrition and increased catabolism (cachexia). Despite the various pathogeneses of the diseases, we were interested in whether the inflammatory process accompanying both diseases has a similar course, and whether inflammatory mediators synthesized inter alia with arachidonic acid will cause a similar cascade of changes in the profile of free fatty acids (FFA). Because both CKD and MetS may have a different etiology, we decided to determine the differences in FA contents between the profiles of patients in both diseases. To date, no studies comparing the qualitative and quantitative changes in FA contents have been performed. Thus, we hope that our study may contribute to the explanation of the mechanisms and the increase of the activity of metabolic processes in both diseases.

## 2. Results

### 2.1. FA in the MetS vs. CG [%]

When comparing the profile of FA between MetS and CG, a significant decrease was determined in the percentage of acids from caprylic (C8:0) to myristylic acid (C14:1) in MetS. The contribution of SFAs were as follows: palmitic acid (C16:0) and its derivative (C16:1) increased with respect to the control group, whereas the percentage of heptadecanoid acid (C17:0), stearic acid (C18:0), and behenic acid (C22:0) as well as lignoceric acid (C24:0) significantly decreased. The percentage of MUFA increases: palmitoleic acid (C16:1), especially oleic acid (C18:1n9ct) and eicosanic acid (C20:1 cis11). Regarding PUFAs, docosatetraenoate (C22:4n6) and docosapentaenate (C22:5w3) but linoleic acid (C18:2n6c) and arachidonic acid (C20:4n6) decreased. A statistically significant, several-fold increase in nervonic acid (C24:1) was also observed, as presented in Table 1.

### 2.2. FA in the CKD vs. CG [%]

When comparing the FA profiles between the CKD and CG groups, a lower amount of caprylic acid (C8:0) was not observed. However, in the case of the capric acid (C10:0) and pentadecanoid acid (C15:0), there was a significant decrease of their percentage amounts in the case of CKD, as compared to the control group, but undecanoic acid (C11:0) and lauric acid (C12:0) in CKD, as compared to the control group, increased. In the case of SFA, the percentage of palmitic acid (C16:0) and its derivative (C16: 1) were significantly higher, and stearic acid (C18:0) was significantly lower, which suggests the activation of the pathway of palmitic acid elongation. Similar to MetS, there was an increase of MUFAs in CKD: myristolenic acid (C14:1), palmitoleic acid (C16:1), oleic acid (C18:1n9 ct), and eicosanic acid (C20:1 cis11). In the CKD group, linoleic acid (C18:2n6c), arachidonic acid (C20:4n6), eicosatrienoic acid (C20:3n3 cis-11), and docosatetraenoate (C22:4n6) were at a similar level, but eicodienoic acid (C20:2 cis11), eicosatrienoic acid (C20:3n6), docosapentaenate (C22:5w3), and DHA (C22:6n3) were significantly higher. Due to the lowered percentage of long-chain FAs such as behenic acid (C22:0), tricosanoic acid (C23:0), and lignoceric acid (C24:0), the amount of accumulated nervonic acid (C24:1) was disturbing and is presented in Table 2.

### 2.3. FA in the CKD vs. MetS [%] and [mg/dL]

**Figure 1:**
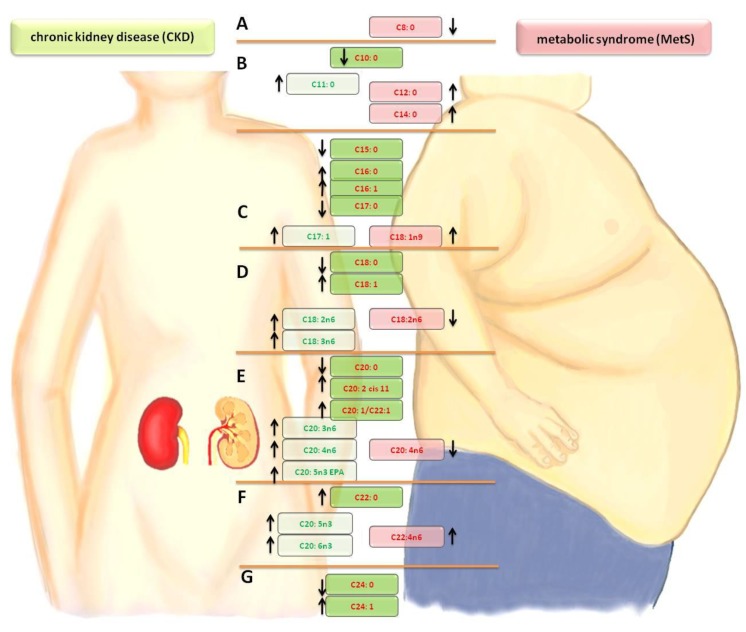
Comparison of changes in the fatty acid profiles in the CKD and MetS groups. A— lowering short-chain FA. B—saturated fatty acid (SFA) elongation. C—mono-unsaturated fatty acid (MUFA) growth at the expense of SFA/palmitic acid derivatives. D—stearic acid derivatives/synthesis of the n-6 family in CKD. E—cascade of arachidonic acid/synthesis of the n-3 and n-6 families. F—increased synthesis of the n-3 and n-6 families. G—synthesis of nervous acid. Arrow- direction of change (increase or decrease in level fatty acid relative to the control group)

Comparing the differences in FA profiles between the groups of patients with MetS and CKD, a significantly lower amount of capric acid (C10:0) was noted in the CKD group. In the case of undecanoic acid (C11:0) to myristic acid (C14:0), an increase of the share in the CKD group was noted. The levels of MUFAs—myristolenic acid (C14:1) and palmitoleic acid (C16:1)—and SFAs —pentadecanoid acid (C15:0), palmitic acid (C16:0) and heptadecanoid acid (C17:0)—were the same in the CKD and MetS groups. The synthesis of oleic acid (C18:1n9 ct) and trans-vaccinic acid (C18:1) was activated in both groups, but it was stronger in the CKD group. The concentration of PUFA—linoleic acid (C18:2n6c)—was reduced, but, for the gamma linoleic acid (C18:3n6), the eicosatrienoic acid (C20:3n6), and the arachidonic acid (C20:4n6), the synthesis was intensified in CKD. The amount of eicosatrienoic acid (C20:3n3 cis-11-) and docosatetraenoate (C22:4n6) in the MetS group and EPA (C20:5n3), docosapentaenate (C22:5w3), and DHA (C22:6n3) in CKD increased. An alarming phenomenon is the high concentration of nervonic acid (C24:1) in both groups (MetS and CKD), which is presented in Table 3. Changes in the fatty acid profiles are shown in Figure 1. When comparing the percentage of fatty acids in the plasma with their amount, it was observed that practically all FAs in patients with kidney disease are elevated in comparison to people with metabolic syndrome. There were also three fatty acids whose percentage amounts significantly correlate with the highest number of parameters in haemodialysis patients. These were oleic acid (C18:1n9), gamma linoleic acid (C18:3n6) and docosatetraenoate acid (C22:4n6), as shown in Table 4.

## 3. Discussion

### 3.1. FA in the MetS vs. CG

Due to the fact that caprylic acid (C8:0) has confirmed antibacterial and antifungal properties, which limits the growth of pathogenic bacteria and fungi in the digestive tract [11,12], its increased concentration might be the effect of the mechanism compensating for more frequent infections and/or the cause of the mechanism buffering the state of overeating. However, it also might be the result of increased intake or several factors at the same time. A similar growing tendency for caprylic acid (C8:0) was observed by other researchers in the group of women with polycystic ovary syndrome who also often suffer from metabolic syndrome [13]. The decreased concentration and percentage of other medium-chain fatty acids (C10:0; C12:0; C14:0; C15:0) may result from the use of these acids for the synthesis of long-chain fatty acids and MUFA [14]. No reduction in the level of palmitic acid (C16:0) was observed, but an increase in palmitoleic acid (C16:1) and elongation into stearic acid (C18:0) were noted. The latter then undergoes desaturation into its metabolites, such as oleic acid (C18:1w9) and vaccenic acid (C18:1 trans11). This synthesis pathway with the use of sterol regulatory element-binding transcription factor 1 (SREBP-1c) is stimulated by high levels of insulin and cholesterol, which accompany the metabolic syndrome [15]. It was determined that saturated fatty acids (SFA) such as C16:0 and C18:0 are responsible for increased lipotoxicity, insulin resistance, and cell death via apoptosis [4]. Similar observations concerning higher concentrations of oleic acid (C18:1w9) in the group of obese women and women with PCOS were made by other researchers [16]. There was an increase in dihomo-γ-linolenic acid (DGLA, C20:3n-6), arachidonic acid (AA, C20:4n-6), and their derivatives, which contribute to the effectiveness of the inflammatory state treatment [17]. The synthesis of arachidonic acid enables the synthesis of prostaglandins (PG) and 2-series thromboxanes (TX) and 4-series leukotrienes, 12, 15 hydroxyeicosatetraenoic acids (HETE) [18]. The mechanisms of these reactions are based on the action of cyclooxygenase (COX) with the formation of PG and TX and lipoxygenase catalyzing the synthesis of the non-cyclic compounds Hydroperoxyeicosatetraenoic acid (HPETE) and HETE. It is known from the literature that these compounds are much more active than the derivatives of DGLA (C20:3n6) and EPA (C20:5n3). In this study, similar to the results shown by Yamazaki et al., the level of long-chain fatty acids decreases, especially behenic acid (C22:0) and lignoceric acid (C24:0) [19]. This suggests the use of these acids for the synthesis of nervonic acid (C24:1), which is present in the myelin sheath of nerve cells and is typical for PCOS [13,20].

### 3.2. FA in the CKD vs. CG

In the group of hemodialysis female patients, the percentage of caprylic acid (C8:0) did not differ from that of the control group. However, similar to the group with metabolic syndrome, the processes of elongation from C10:0 to C16:0 and de-saturated derivative C16:1 were intensified. The decrease in the percentage of stearic acid (C18:0) confirms the enhanced synthesis of its derivatives, C18:1n9 and C20:1, which are still lower than in MetS. It seems that the further elongation of SFA is also the cause of an increased level of lignoceric acid (C24:0), which was not observed because it underwent a more intensive desaturation into nervonic acid (C24:1) in both diseases. In addition, a more intensive synthesis of arachidonic acid (C20:4n6) was observed from essential fatty acids (EFAs), which probably includes linoleic acid (C18:2n6). These derivatives are responsible for the formation of blood clots, atherosclerotic changes, inflammatory and allergic reactions, cell proliferation, and the development of tumor tissue [21]. An increased percentage of C22:4n6 and omega-3-family acids, including C22:5n3 and C22:6n3 (EPA and DHA), were observed as well. Therefore, the changes undergone in the group of patients with CKD are much more advanced than in the group with metabolic syndrome.

### 3.3. FA in the CKD vs. MetS

When comparing the percentage content of FFA, there was a significant reduction in the concentration of short-chain fatty acids, which include caprylic acid (C8:0) in the MetS group and capric acid (C10:0) in the CKD group. Moreover, in the MetS group, C11:0 to C14:0 levels are reduced in comparison to the control group because they are used for elongation and the acquisition of medium-chain FAs. This may be the result of the use of short-chain fatty acids for energetic purposes due to disturbed glucose metabolism in MetS. Likely due to the decomposition of the fatty tissue and the release of triglycerides and/or the increased expression of palmityl CoaA synthetase and diet, there was an increased concentration of palmitic acid (C16:0) among patients with CKD and MetS. This state suggests a higher degree of chronic inflammatory processes in the group of female patients with kidney diseases, which was described by other authors [22,23]. Podocytes are susceptible to palmitic acid (C16:0), which causes podocyte apoptosis by activating complex 1 of mTOR kinase (mTORC1) signalling. However, it was determined that oleic acid and EPA inhibit the apoptosis induced by palmitate [24]. The expression of stearoyl-CoA desaturase (SCD) is likely a part of a defense mechanism against SFAs and their toxic metabolites [3]. Therefore, the activation of the synthesis of oleic acid (C18:1n9 ct) and trans-vaccinic acid (C18:1) seems to be a protective mechanism in kidney diseases. Moreover, PUFA n-3 effectively reduces inflammatory responses by decreasing or inhibiting the expression of pro-inflammatory cascade compounds [25]. The activation of the enhanced synthesis of EPA and DHA is especially pronounced in CKD. Another observed effect in CKD is the reduction in EFAs, linoleic acid (C18:2n6c), and gamma linoleic acid (C18:3n6), which may be attributed to the use of these fatty acids and the attempt to supress the inflammatory reaction by the synthesis of C22:4n6. Gamma-linolenic acid (C18:3n-6) is a precursor to other acids from the ɷ-6 family, such as eicosatrienoic acid (C20:3n6) and arachidonic acid (C20:4n6), whose synthesis is enhanced both in MetS and CKD. Even though this synthesis is more intensive in MetS, we observe enhanced elongation and an increased level of C22:4n6 in CKD. When analyzing the changes undergone in CKD, one should also remember that the average treatment time of examined female patients with CKD has been 52.125 months. Therefore, the described changes may be appropriate for this period of treatment and may be more or less pronounced in the earlier or later course of CKD, which may be elucidated by limited research.

In our opinion, in MetS, there is also an activation of auxiliary metabolic pathways for oleic acid, which simultaneously inhibit the synthesis of EPA and DHA from ALA. On the other hand, in the group of patients with CKD, we observe a significantly increased synthesis of EPA and DHA. Therefore, it seems that the synthesis of C20:4n6 derivatives is lower in CKD than in MetS because most of this fatty acid is elongated to C22:4n6 in CKD. Other researchers also observed this mechanism [26]. The fatty acids of the n-3 PUFA family are incorporated into the membranes of skeletal muscles, which have anti-inflammatory and anabolic activity. It is assumed that they protect against sarcopenia in CKD, which has been regarded as an independent risk factor of patient death [27]. High levels of arachidonic (C20:4n6) and γ-linolenic (C18:3n3) FFA were linked to a higher percentage of graft survival rate [28]. Moreover, γ-linolenic acid (GLA, C18:3n-6) is the only member of the n-6 fatty acid family, which has anti-carcinogenic activity [29]. Its concentration increases in hemodialysis female patients due to the use of the precursor in a derivative formation. Thus, we do not know whether the production of C18:3n-6 derivatives from GLA increases the survival rate or whether it signifies a more advanced inflammatory process, which would reduce the survival rate. It should be noted that there were significant differences between the amount of almost all FAs in female patients with kidney diseases. In our opinion, this was related not only to diet but also to the mobilization of fatty acids from the adipose tissue in patients with progressing malnutrition or organism wasting. Moreover, observed correlations between oleic acid (C18:1n9) and the parameters of patients indicated better nutrition of the patients and less advanced disease. However, the increase in γ-linolenic acid (GLA, C18:3n-6) is connected to height, period of treatment, and the date of first dialysis, in contrast to docosatetraenoate acid (C22:4n6), which was correlated only with the date of the first dialysis and the period of treatment. An observation arises that the height of a patient and progressive kidney failure will be characterized by higher concentrations of docosatetraenoate acid (C22:4n6). In both diseases, MetS and CKD, an increase in nervonic (C24:1) acid was observed, which was also noted in other diseases, such as multiple sclerosis (MS). MS is an inflammatory disease characterized by central demyelination and axons loss [30]. Yamazaki linked nervonic acid to metabolic syndrome, and numerous studies suggest its relationship with cardiovascular risk, neuron disintegration, and the use of C24:1 as a necrosis marker. This study showed a large increase in nervonic acid in the CKD group and the MetS group. We expected the increase in the level of this acid in CKD patients, which was suggested by the study of Shearer et al. The increase was not several-fold higher in comparison with the MetS group, but was statistically significant when compared to the control group [7]. The study seems to be limited by the degree of progression of metabolic syndrome and its accompanying diseases. The measurement of fatty acids itself is a demanding and costly task. This is why its availability for healthcare facilities is low at this stage.

## 4. Materials and Methods

The Ethics Committee NKEBN/417/2015-ST-48 approved the study protocol on October 24 2015. All patients gave written informed consent and their confidentiality and anonymity were protected. The study involved 73 Caucasian women. The first test group included 24 women with chronic kidney disease (CKD) at the age of 66.7 ± 13.04 years and with an average body weight of 67.09 ± 18.93 kg. Other parameter characteristics for the profile of this disease are presented in Table 5. The second test group comprised of 19 women at the age of 60.32 ± 7.64 and with an average body weight of 81.37 ± 11.11 kg with metabolic syndrome. Other parameters typical for the profile of this disease are presented in Table 2. The third group, the control group, included 30 healthy women at the age of 58.23 ± 8.6 years and with a body weight of 59.87 ± 5.08 kg. In the control group, all the women had a proper body weight with respect to their height. The characteristics of this group are presented in Table 6.

### 4.1. Isolation of Fatty Acids

We obtained plasma from whole blood samples collected in tubes containing EDTA as an anticoagulant by centrifugation for 10 min at 1200 G. Plasma samples were stored at −80 °C. Fatty acids were extracted according to the Folch method and analyzed by gas chromatography. Plasma (0.5 mL) was saponified with 1 mL of 2 mol/L KOH methanolic solution at 700 °C for 20 min and then methylated with 2 mL of 14% boron trifluoride in methanol under the same conditions. Then, 2 mL of n-hexane and 10 mL of saturated NaCl solution were added. One milliliter of the n-hexane phase was collected for analysis.

### 4.2. Analysis of Fatty Acid Methyl Esters

Gas chromatography was performed using the Agilent Technologies 7890A GC System (SUPELCOWAX™ 10) Capillary GC Column (15 mm × 0.10 mm, 0.10 μm, Supelco, Bellefonte, PA, USA). Chromatographic conditions were as follows: the initial temperature was 40 °C for 0.5 min, and then it increased at a rate of 25 °C/min to 195 °C (0 min). Next, it was increased at a rate of 3 °C/min to 205 °C (0 min), and then increased at a rate of 8 °C/min to 250 °C for 0.5 min. The total analysis lasted approximately 16.16 min and the gas flow rate was 1 mL/min with hydrogen as the carrier gas. Fatty acids were identified by comparing their retention times with those of commercially available standards. The analysis of fatty acid contents in particular samples was performed using specialist ChemStation Software (Agilent Technologies Inc, London, UK). The qualitative identification of fatty acids was performed by comparing retention times to those of the standards, and the quantity of a specific fatty acid was given as a percentage of the fatty acid in the total amount of analyzed compounds (excluding the standard, heneicosanoic acid (C21:0)).

### 4.3. Statistical Analysis

Statistical analyses were performed using Statistica 12.0 software (Statsoft, Tulsa, OK, USA). The results are expressed as mean ± standard deviation. Since the distribution in most cases is deviated from normal conditions (Shapiro–Wilk test), a non-parametric Mann–Whitney test was used for comparisons between the groups. The statistical significance was shown in two levels: the first level was *p* < 0.05 and the second level was *p* **<** 0.01. In order to evaluate the correlation between variables, the Spearman rank correlation test was used. To observe the proportional changes of the fatty acids, the results were also given as their percentages in blood plasma, where 100% was the amount of fatty acids in a given group.

## 5. Conclusions

There is a lower amount of C10:0 in CKD than MetS, whereas there are more saturated fatty acids, C11:0-C14:0, in CKD than in MetS. The activation of the synthesis of oleic acid (C18:1n9 ct) and trans-vaccinic acid (C18:1) is likely a defense mechanism in CKD and MetS due to the increased amount of SFA in blood plasma. The cause of the increased amount of all FAs in blood plasma, especially in the case of palmitic (C16:0) and stearic (C18:0) acids, may be—besides diet—the decomposition of adipose tissue and the progressing devastation of the organism in CKD. However, in MetS, the main reason may be the intake of diet. Higher concentrations of palmitic (C16:0) and the expenditure of stearic (C18:0) fatty acids may be a marker of the progressing organism wasting in the course of kidney failure, but a better marker may be the increase in the level of docosatetraenoate acid (C22:4n6). The increase in nervonic acid (C24:1) is more visible in the CKD group than in the MetS group. The increase in nervonic acid (C24:1) is more visible in the CKD group than in the MetS group, which suggests a higher degree of demyelination and loss of axons.

## Figures and Tables

**Table 1:** Comparison of the contents of fatty acid (FA) in metabolic syndrome (MetS) vs. the control group (CG) [%]. EPA: eicosapentanoic acid. DHA: docosahexaenoic acid.

Fatty Acids	MetS			CG			*p*
	Median	MEAN	SD	Median	MEAN	SD	
C8:0 Caprylic acid	0.00	0.000	0.000	0.06	0.035	0.035	*p* < 0.01
C10:0 Capric acid	0.82	0.668	0.434	7.27	4.015	5.473	*p* < 0.05
C11:0 Undecanoic acid	0.01	0.023	0.013	0.06	0.036	0.035	*p* = 0.17
C12:0 Lauric acid	0.06	0.145	0.155	0.20	0.221	0.152	*p* < 0.01
C14:0 Myristic acid	0.38	1.175	0.325	0.48	1.442	0.421	*p* < 0.01
C14:1 Myristolenic acid	0.04	0.074	0.049	0.06	0.044	0.070	*p* < 0.01
C15:0 Pentadecanoid acid	0.21	0.333	0.114	0.24	0.396	0.156	*p* = 0.23
C16:0 Palmitic acid	1.28	27.905	1.734	4.12	25.423	2.363	*p* < 0.01
C16:1 Palmitoleic acid	0.80	1.944	0.510	0.70	1.460	0.487	*p* < 0.01
C17:0 Heptadecanoid acid	0.04	0.349	0.041	0.07	0.476	0.122	*p* < 0.01
C17:1 cis-10- Heptadecanoid acid	0.10	0.154	0.149	0.11	0.134	0.076	*p* = 0.41
C18:0 Stearic acid	1.27	11.500	0.962	2.13	13.319	1.791	*p* < 0.01
C18:1n9 ct Oleic acid	3.23	23.283	2.543	3.34	17.661	3.152	*p* < 0.01
C18:1 Trans-vaccinic acid	0.16	2.028	0.177	0.32	1.611	0.276	*p* < 0.01
C18:2n6c Linoleic acid	3.51	17.356	2.782	2.91	20.877	1.962	*p* < 0.01
C18:3n6 Gamma linoleic acid	0.12	0.212	0.071	0.14	0.230	0.133	*p* = 0.56
C18:3n3 Linolenic acid	0.30	0.676	0.255	0.28	0.614	0.208	*p* = 0.55
C20:0 Arachidic acid	0.05	0.156	0.041	0.14	0.221	0.159	*p* < 0.05
C22:1/C20:1 cis11- eicosanic acid	0.10	0.268	0.172	0.20	0.156	0.120	*p* < 0.05
C20:2 cis-11-eicodienoic acid	0.03	0.211	0.043	0.18	0.103	0.091	*p* < 0.01
C20:3n6 Eicosatrienoic acid	0.30	1.130	0.285	0.40	1.220	0.336	*p* = 0.32
C20:4n6 Arachidonic acid	1.54	5.410	1.052	1.12	5.908	1.328	*p* = 0.43
C20:3n3 cis-11-eicosatrienoic acid	0.03	0.010	0.016	0.00	0.000	0.000	*p* < 0.01
C20:5n3 Eicosapentanoic acid	0.56	0.913	0.521	0.86	1.096	1.031	*p* = 0.22
C22:0 Behenic acid	0.03	0.050	0.037	0.27	0.167	0.243	*p* < 0.05
C22:1n9 13 Erucic acid	0.02	0.577	1.730	0.00	0.025	0.050	*p* = 0.12
C23:0 Tricosanoic acid	0.08	0.130	0.247	0.25	0.133	0.128	*p* = 0.76
C22:4n6 (docosatetraenoate)	0.21	0.266	0.162	0.15	0.097	0.071	*p* < 0.01
C22:5w3 (docosapentaenate)	0.10	0.591	0.154	0.28	0.504	0.234	*p* = 0.32
C24:0 Lignoceric acid	0.38	0.075	0.169	0.00	0.307	0.708	*p* < 0.05
C22:6n3 Decosahexaenoic acid	0.48	2.177	0.536	1.19	1.842	1.257	*p* = 0.19
C24:1 Nervonic acid	0.04	0.055	0.043	0.00	0.009	0.033	*p* < 0.01

**Table 2:** Comparison of the content of FAs in chronic kidney disease (CKD) vs. CG [%].

Fatty Acids	CKD			CG			*p*
	Median	MEAN	SD	Median	MEAN	SD	
	0.07	0.042	0.037	0.06	0.035	0.035	*p* = 0.30
C10:0 Capric acid	0.03	0.017	0.019	7.27	4.015	5.473	*p* < 0.05
C11:0 Undecanoic acid	0.02	0.074	0.014	0.06	0.036	0.035	*p* < 0.01
C12:0 Lauric acid	0.05	0.210	0.143	0.20	0.221	0.152	*p* < 0.05
C14:0 Myristic acid	0.35	1.370	0.316	0.48	1.442	0.421	*p* = 0.21
C14:1 Myristolenic acid	0.06	0.073	0.039	0.06	0.044	0.070	*p* < 0.01
C15:0 Pentadecanoid acid	0.09	0.292	0.084	0.24	0.396	0.156	*p* < 0.01
C16:0 Palmitic acid	1.18	28.042	0.954	4.12	25.423	2.363	*p* < 0.01
C16:1 Palmitoleic acid	1.19	2.130	0.700	0.70	1.460	0.487	*p* < 0.01
C17:0 Heptadecanoid acid	0.09	0.377	0.076	0.07	0.476	0.122	*p* < 0.01
C17:1 cis-10- Heptadecanoid acid	0.06	0.191	0.050	0.11	0.134	0.076	*p* < 0.05
C18:0 Stearic acid	1.30	10.770	0.760	2.13	13.319	1.791	*p* < 0.01
C18:1n9 ct Oleic acid	1.44	20.605	1.402	3.34	17.661	3.152	*p* < 0.01
C18:1 Trans-vaccinic acid	0.25	1.845	0.173	0.32	1.611	0.276	*p* < 0.01
C18:2n6c Linoleic acid	4.56	19.358	2.468	2.91	20.877	1.962	*p* = 0.34
C18:3n6 Gamma linoleic acid	0.09	0.344	0.070	0.14	0.230	0.133	*p* < 0.01
C18:3n3 Linolenic acid	0.22	0.712	0.201	0.28	0.614	0.208	*p* = 0.45
C20:0 Arachidic acid	0.04	0.097	0.025	0.14	0.221	0.159	*p* < 0.01
C22:1/C20:1 cis11- eicosanic acid	0.14	0.334	0.331	0.20	0.156	0.120	*p* < 0.01
C20:2 cis-11-eicodienoic acid	0.04	0.186	0.029	0.18	0.103	0.091	*p* < 0.01
C20:3n6 Eicosatrienoic acid	0.63	1.689	0.334	0.40	1.220	0.336	*p* < 0.01
C20:4n6 Arachidonic acid	1.90	6.488	1.192	1.12	5.908	1.328	*p* = 0.17
C20:3n3 cis-11-eicosatrienoic acid	0.00	0.000	0.000	0.00	0.000	0.000	*p* = 1
C20:5n3 Eicosapentanoic acid	0.88	1.279	0.721	0.86	1.096	1.031	*p* < 0.05
C22:0 Behenic acid	0.07	0.024	0.029	0.27	0.167	0.243	*p* < 0.05
C22:1n9 13 Erucic acid	0.00	0.000	0.000	0.00	0.025	0.050	*p* = 0.23
C23:0 Tricosanoic acid	0.10	0.070	0.071	0.25	0.133	0.128	*p* = 0.12
C22:4n6 (docosatetraenoate)	0.10	0.127	0.087	0.15	0.097	0.071	*p* = 0.22
C22:5w3 (docosapentaenate)	0.20	0.682	0.148	0.28	0.504	0.234	*p* < 0.05
C24:0 Lignoceric acid	0.02	0.010	0.020	0.00	0.307	0.708	*p* = 0.32
C22:6n3 Decosahexaenoic acid	1.30	2.487	0.714	1.19	1.842	1.257	*p* < 0.05
C24:1 Nervonic acid	0.08	0.062	0.044	0.00	0.009	0.033	*p* < 0.01

**Table 3:** Comparison of the amount of FA in CKD vs MetS [%].

Fatty Acids	CKD			MetS			*p*
	Median	MEAN	SD	Median	MEAN	SD	
C8:0 Caprylic acid	0.07	0.042	0.037	0.00	0.000	0.000	*p* < 0.01
C10:0 Capric acid	0.03	0.017	0.019	0.82	0.668	0.434	*p* < 0.01
C11:0 Undecanoic acid	0.02	0.074	0.014	0.01	0.023	0.013	*p* < 0.01
C12:0 Lauric acid	0.05	0.210	0.143	0.06	0.145	0.155	*p* < 0.01
C14:0 Myristic acid	0.35	1.370	0.316	0.38	1.175	0.325	*p* < 0.01
C14:1 Myristolenic acid	0.06	0.073	0.039	0.04	0.074	0.049	*p* = 0.87
C15:0 Pentadecanoid acid	0.09	0.292	0.084	0.21	0.333	0.114	*p* = 0.34
C16:0 Palmitic acid	1.18	28.042	0.954	1.28	27.905	1.734	*p* = 0.28
C16:1 Palmitoleic acid	1.19	2.130	0.700	0.80	1.944	0.510	*p* = 0.36
C17:0 Heptadecanoid acid	0.09	0.377	0.076	0.04	0.349	0.041	*p* = 0.22
C17:1 cis-10- Heptadecanoid acid	0.06	0.191	0.050	0.10	0.154	0.149	*p* < 0.01
C18:0 Stearic acid	1.30	10.770	0.760	1.27	11.500	0.962	*p* < 0.05
C18:1n9 ct Oleic acid	1.44	20.605	1.402	3.23	23.283	2.543	*p* < 0.01
C18:1 Trans-vaccinic acid	0.25	1.845	0.173	0.16	2.028	0.177	*p* < 0.01
C18:2n6c Linoleic acid	4.56	19.358	2.468	3.51	17.356	2.782	*p* = 0.29
C18:3n6 Gamma linoleic acid	0.09	0.344	0.070	0.12	0.212	0.071	*p* < 0.01
C18:3n3 Linolenic acid	0.22	0.712	0.201	0.30	0.676	0.255	*p* = 0.40
C20:0 Arachidic acid	0.04	0.097	0.025	0.05	0.156	0.041	*p* < 0.01
C22:1/C20:1 cis11- eicosanic acid	0.14	0.334	0.331	0.10	0.268	0.172	*p* = 0.28
C20:2 cis-11-eicodienoic acid	0.04	0.186	0.029	0.03	0.211	0.043	*p* < 0.01
C20:3n6 eicosatrienoic acid	0.63	1.689	0.334	0.30	1.130	0.285	*p* < 0.01
C20:4n6 Arachidonic acid	1.90	6.488	1.192	1.54	5.410	1.052	*p* = 0.19
C20:3n3 cis-11-eicosatrienoic acid	0.00	0.000	0.000	0.03	0.010	0.016	*p* < 0.01
C20:5n3 Eicosapentanoic acid	0.88	1.279	0.721	0.56	0.913	0.521	*p* < 0.01
C22:0 Behenic acid	0.07	0.024	0.029	0.03	0.050	0.037	*p* = 0.21
C22:1n9 13 Erucic acid	0.00	0.000	0.000	0.02	0.577	1.730	*p* < 0.01
C23:0 Tricosanoic acid	0.10	0.070	0.071	0.08	0.130	0.247	*p* = 0.17
C22:4n6 (docosatetraenoate)	0.10	0.127	0.087	0.21	0.266	0.162	*p* < 0.01
C22:5w3 (docosapentaenate)	0.20	0.682	0.148	0.10	0.591	0.154	*p* = 0.66
C24:0 Lignoceric acid	0.02	0.010	0.020	0.38	0.075	0.169	*p* < 0.01
C22:6n3 Decosahexaenoic acid	1.30	2.487	0.714	0.48	2.177	0.536	*p* = 0.22
C24:1 Nervonic acid	0.08	0.062	0.044	0.04	0.055	0.043	*p* = 0.47

**Table 4:** Correlation matrix for selected FA with parameters of CKD patients. iPTH: parathyroid hormone. CREA: creatinine. HGB: hemoglobin. FER: ferritin. ALB: albumin. Kt/V: dialysis adequacy.

Parameters	C18:1n9 ct Oleic Acid	C18:3n6 Gamma Linoleic Acid	C22:4n6 (Docosatetraenoate)
Age (years)	0.489 *	−0.116	−0.502 *
Ca (mg/dL)	−0.396	−0.104	0.546 *
Phosphorus (mg/dL)	−0.089	0.217	0.049
iPTH (pg/Ml)	−0.288	−0.202	0.339
CREA (mg/dL)	−0.491	0.603	0.203
HGB (g/Dl)	−0.469 *	0.019	0.269
% Transferrin saturation (%)	0.278	−0.136	−0.155
FER (ug/L)	0.224	−0.116	−0.318
ALB (g/L)	−0.369	−0.072	0.520
K (mEq/L)	−0.226	0.091	0.298
Height (cm)	0.039	0.514 *	−0.420 *
Date of the first dialysis	0.236	0.552 *	−0.448 *
Weight (kg)	0.448 *	0.330	−0.223
Time of treatment (months)	−0.236	−0.552 *	0.448 *
BMI (kg/m^2^)	0.473 *	0.218	−0.146
Body surface (m^2^)	0.390	0.430 *	−0.293
Kt/V	−0.504 *	−0.096	0.329
Clearance (ml/min)	−0.313	0.427 *	0.119
nPCR (g/kg/day)	−0.082	0.146	0.090
Ca × P (mg^2^/dL^2^)	−0.302	0.110	0.366

* Significant correlation.

**Table 5:** The first test group includes patients suffering from chronic kidney disease (CKD).

Characteristic	Avg ± SD
Age (years)	66.71 ± 13.04
Weight (kg)	67.09 ± 18.93
BMI (kg/m^2^)	23.97 ± 6.39
Ca (mg/dL)	8.664 ± 1.36
I PHOS (mg/dL)	5.395 ± 1.16
iPTH (pg/mL)	1058.57 ± 842.62
CREA (mg/dL)	5.72 ± 1.53
HGB (g/dL)	10.76 ± 1.04
%TSAT (%)	27.92 ± 14.69
FER (ug/L)	839.75 ± 645.03
ALB (g/L)	36.92 ± 4.06
K (mEq/L)	5.446 ± 0.65
Time of treatment (months)	52.125 ± 46.64
Body surface (m^2^)	1.705 ± 0.22
Kt/V	1.788 ± 0.33
Clearance (mL/min)	236.11 ± 43.23
nPCR (g/kg/day)	1.077 ± 0.26
Ca x P (mg^2^/dL^2^)	46.875 ± 10.21

**Table 6:** Clinical and biochemical data of patients with metabolic syndrome (MetS) and the control group (CG).

Characteristic	MetS avg ± SD	CG avg ± SD	*p*-Value
Age (years)	60.32 ± 7.34	58.23 ± 8.6	0.0627
Weight (kg)	81.37 ± 11.1 *	59.87 ± 5.08 *	0.0003
BMI (kg/m2)	30.51 ± 2.02 *	22.86 ± 1.35 *	0.0002
WHR	0.94 ± 0.05 *	0.78 ± 0.082 *	0.0481
Glucose (mg/dL)	116.06 ± 35.71	87.65 ± 13.68 *	0.0027
Uric acid (mg/dL)	6.02 ± 1.09	5.23 ± 0.7	0.0574
Cholesterol (mg/dL)	218.4 ± 30.33	186.7 ± 22.2	0.0372
LDL (mg/dL)	118.87 ± 25.67 *	91.2 ± 14.6 *	0.0500
TRIGL (mg/dL)	154.26 ± 28.84 *	98.8 ± 35.8 *	0.0000
HDL (mg/dL)	58.67 ± 15.03	62.1 ± 10.8	0.0621

* Statistically significant differences *p* < 0.05.

## Abbreviations

ALA	Alpha lipoic acid
ALB	Albumin
CG	Control group
CKD	Chronic kidney disease
CREA	Creatinine
C18:3n6/C22:4n6	Gamma linoleic acid/ docosatetraenoate
DHA	Docosahexaenoic acid
EPA	Eicosapentanoic acid
FA	Fatty acid
FER	Ferritin
GFR	Glomerular filtration rate
GLA	Gamma-linolenic acid
HGB	Haemoglobin
iPTH	Parathyroid hormone
Kt/V	Dialysis adequacy
LA	Linoleic acid
MetS	Metabolic syndrome
MUFA	Mono-unsaturated fatty acids
NAFLD	Non-alcoholic fatty liver disease
PUFA	Poly-unsaturated fatty acids
SFA	Saturated fatty acids
